# Oral Chemotherapy for Treatment of Lung Cancer

**DOI:** 10.3389/fonc.2020.00793

**Published:** 2020-04-28

**Authors:** Sushma Jonna, Joshua E. Reuss, Chul Kim, Stephen V. Liu

**Affiliations:** ^1^Lombardi Comprehensive Cancer Center, Georgetown University, Washington, DC, United States; ^2^Johns Hopkins Sidney Kimmel Comprehensive Cancer Center, Baltimore, MD, United States

**Keywords:** oral therapy, chemotherapy, capecitabine, temozolomide, topotecan, vinorelbine, etoposide

## Abstract

The global COVID-19 pandemic has disrupted healthcare delivery, particularly for patients with advanced lung cancer. While certain aspects of care can be safely omitted or delayed, systemic therapy plays an important role in survival and quality of life for patients with advanced lung cancer; limiting access to systemic therapy will compromise cancer-related outcomes. This can be at odds with strategies to mitigate risk of COVID-19 exposure, which include reducing hospital and clinic visits. One important strategy is implementation of oral cancer therapies. Many standard regimens require intravenous infusions but there are specific circumstances where an oral agent could be an acceptable alternative. Integrating oral therapeutics can permit patients to receive effective systemic treatment without the exposure risks associated with frequent infusions. Here, we review currently available oral cytotoxic agents with a potential role in the treatment of lung cancer.

## Introduction

The global pandemic of the novel severe acute respiratory syndrome coronavirus 2 (SARS-CoV-2), or coronavirus disease (COVID-19), has influenced every aspect of health care, particularly for patients with cancer. Early reports suggest that patients with cancer, particularly lung cancer, may be at higher risk for contracting COVID-19 (1, 2). An analysis of patients with cancer treated at the Zhongnan Hospital in Wuhan, China revealed a higher COVID-10 infection rate than the general Wuhan population (0.79 vs. 0.37%) (3). The majority of COVID-19 infected patients from that cancer center, however, were not receiving any active therapy, suggesting a possible risk was simply exposure to the cancer center itself. Efforts to reduce the risk of COVID-19 exposure have led to a reshaping of cancer care delivery, largely focused on reducing hospital and clinic visits with the use of telemedicine (4). A complementary strategy for patients with advanced cancer requiring systemic therapy is the use of oral therapeutics in lieu of intravenous agents. While not always possible, when a suitable oral equivalent is available, its use can reduce clinic visit frequency and potentially COVID-19 exposure. For patients with alterations in driver oncogenes, use of oral tyrosine kinase inhibitors is already standard practice (5). For patients without actionable driver alterations, oral treatment options are less readily available, but there are several oral cytotoxic agents (Table 1) which can easily be implemented in an effort to reduce the risk of exposures in the wake of the COVID-19 pandemic. In this review, we will summarize the data for oral chemotherapeutics in lung cancer and discuss the utility of these agents in the outpatient management of advanced disease.

**Table 1:** Available formulations of oral chemotherapeutics.

**Agent**	**Trade name**	**Capsule dosage strength**
Etoposide	VePesid®	Capsules: 50 mg (6)
Topotecan	Hycamptin®	Capsules: 0.5, 1 mg (7)
Temozolomide	Temodar®	Capsules: 5, 20, 100, 140, 180, 250 mg (8)
Vinorelbine	Navelbine®	Capsules: 20, 30 mg (9)
Tegafur/gimeracil/oteracil (S-1)	Teysuno®	Capsules: 15 mg/4.35 mg/11.8 mg (10)
Capecitabine	Xeloda®	Tablets: 150, 500 mg (11)

### Etoposide

Etoposide, a cytotoxic agent that inhibits topoisomerase II, is widely used across tumor types. The combination of platinum and etoposide chemotherapy has been the front-line therapy for the treatment of small cell lung cancer (SCLC) for decades. While the addition of anti-PD-L1 antibodies (atezolizumab, durvalumab) to first-line therapy has improved survival, the platinum plus etoposide backbone remains central to the treatment of this disease (12, 13). Platinum agents (and the anti-PD-L1 antibodies) are delivered intravenously (IV), but etoposide is available in both IV and oral formulations. A prospective phase II trial randomized 83 patients with SCLC to a standard IV regimen (cisplatin 100 mg/m^2^ IV on day 1 plus etoposide 120 mg/m^2^ IV days 1–3) or a hybrid IV and oral regimen (cisplatin 100 mg/m^2^ IV on day 1, etoposide 120 mg/m^2^ IV on day 1 and etoposide 240 mg/m^2^ orally on days 2 and 3), both in 28-day cycles (14). There was no significant difference in overall response rate (RR) between the oral and IV regimens (50 vs. 59%, *p* = 0.438). PFS and OS were also comparable in both treatment arms. Most toxicities, including grade 3–4 hematologic toxicity, alopecia, nausea, vomiting, and diarrhea were similar in both groups. Infectious episodes, moderate to severe anemia, and weight loss occurred more frequently with the IV regimen. Other trials with different dosing schedules for oral etoposide, including 5- or 21-day regimens, also provided comparable outcomes to IV regimens (15, 16).

The cisplatin plus etoposide IV-oral hybrid regimen was also used as a standard arm in several other SCLC clinical trials and performed well. A large (*n* = 436) randomized trial showed superiority of platinum plus etoposide over CEV (cyclophosphamide, epirubicin, and vincristine) (17). The standard arm utilized oral etoposide (cisplatin 75 mg/m^2^ on day 1, etoposide 100 mg/m^2^ IV on day 1 and etoposide 200 mg/m^2^ orally on days 2–4 every 3 weeks) and included 214 patients with limited stage (LS)-SCLC (who received concurrent thoracic radiation therapy). Median survival was longer with platinum plus etoposide (14.5 months) compared to CEV (9.7 months) among patients with LS-SCLC (*p* = 0.001). For patients with extensive stage (ES)-SCLC, survival was equivalent between the two arms. While the chemo-immunotherapy regimens employing atezolizumab and durvalumab, studied in IMpower133 and CASPIAN, respectively, only used IV etoposide, extrapolation to a hybrid IV-oral regimen could be a reasonable option to reduce infusion center visits and potential COVID-19 exposure (12, 13).

Data exploring use of oral etoposide in non-small cell lung cancer (NSCLC) is sparse, primarily consisting of early phase studies done in the 1990s prior to the development of modern platinum-doublet regimens. In phase II trials in treatment-naïve advanced NSCLC, oral etoposide monotherapy at a dose of 50 mg/m^2^ for 21 days every 4 weeks offered a RR of 7% to 26% (18–20). Based on the advantages reported in SCLC, the efficacy of combination therapies with oral etoposide and IV platinum agents was evaluated but these combinations offered modest outcomes and were replaced by more modern chemotherapy doublets (21). While a viable option for clinical use in SCLC, there is a limited role for oral etoposide in patients with NSCLC.

### Topotecan

The topoisomerase I inhibitor topotecan is the current standard of care, and the only FDA-approved therapy, for patients with relapsed SCLC. It is available in oral formulations that provide comparable efficacy and toxicity to the IV form. A randomized phase II trial enrolled 106 patients with relapsed SCLC and randomized them to topotecan orally at 2.3 mg/m^2^/day or topotecan IV at 1.5 mg/m^2^/day, both for 5 days in 21-day cycles (22). The RR was comparable between oral and IV formulations (23 and 15%, respectively) with similar durations of response (18 and 14 weeks). Both regimens improved symptoms. Median OS was 32 weeks with oral topotecan and 25 weeks with IV topotecan. Grade 3–4 thrombocytopenia and anemia were similar between the two arms, though grade 4 neutropenia was less common with oral topotecan (35.3%) compared to IV topotecan (67.3%).

A phase III trial confirmed the activity of oral topotecan in SCLC. Patients with SCLC (*n* = 309) who had relapsed after an initial response to platinum-based chemotherapy were randomized to topotecan orally at 2.3 mg/m^2^/day or topotecan IV at 1.5 mg/m^2^/day on days 1–5 in 21-day cycles (23). The RR was 18% with oral and 22% with IV topotecan. Median time to progression (TTP) was similar between oral and IV formulations (12 and 14.6 weeks) as was median OS (33 and 35 weeks, respectively). Similar to the phase II study, toxicity was similar in the two arms, though grade 4 neutropenia was more common with IV topotecan (64.2%) than oral topotecan (47%) and grade 4 thrombocytopenia was more common with the oral regimen (28.7 vs. 18%). A phase I study explored weekly dosing of oral topotecan, starting at 3 mg/m^2^ on days 1, 8, and 15 in 28-day cycles and escalating by 0.5 mg/m^2^ (24). The maximum tolerated dose was 4 mg/m^2^ with this weekly regimen. At that dose level, grade 3–4 neutropenia and anemia were seen in 7.1% of patients, and there was no grade 3 or higher thrombocytopenia. Larger studies using this approach are lacking.

Though not approved, there is also evidence showing activity of oral topotecan in NSCLC. A phase II trial of oral topotecan 2.3 mg/m^2^/day on days 1–5 every 21 days was completed in 30 patients with previously untreated advanced NSCLC (25). No responses were observed, though 13 patients achieved stable disease and the median TTP was 12.3 weeks. Median OS was 39.9 weeks with a 1-year survival rate of 33.3%. Grade 3–4 neutropenia was noted in 40% of patients. This led to a phase III randomized trial comparing oral topotecan 2.3 mg/m^2^/day (given for 5 consecutive days) with standard IV docetaxel 75 mg/m2, both in 21-day cycles (26). This non-inferiority trial included 829 patients with previously treated NSCLC. The 1-year survival rate was 25.1% with oral topotecan vs. 28.7% with docetaxel, meeting the defined endpoint of non-inferiority of oral topotecan to docetaxel. RR was low (5%) in both groups with a median TTP of 11.3 weeks with topotecan and 13.1 weeks with docetaxel. The median OS was 27.9 weeks with topotecan and 30.7 weeks with docetaxel (*p* = 0.57). Hematologic toxicities were similar to other trials, with grade 3–4 anemia and thrombocytopenia noted in 26% of patients receiving topotecan.

Alternate dosing schedules have also been explored in NSCLC with similar results. A phase II study randomized 80 patients with NSCLC to second line oral topotecan at 2–2.5 mg daily (fixed dose) for 5 of 7 days given 2 of 3 weeks or standard IV docetaxel 75 mg/m^2^ every 21 days (27). Outcomes were comparable between the two arms. The RR with oral topotecan was 8% with a median TTP of 1.6 months, a median OS of 8.4 months and a 1-year survival rate of 36%. Topotecan was associated with grade 3–4 neutropenia (18%), anemia (13%), and thrombocytopenia (24%).

Overall, studies support equivalent efficacy and similar toxicity for oral vs. IV topotecan as 2nd line therapy for patients with relapsed SCLC. Oral topotecan is approved and available for use in this indication. It has limited efficacy in NSCLC but is an orally bioavailable option which may be appealing in specific clinical circumstances.

### Temozolomide

Temozolomide (TMZ) is an orally bioavailable alkylating agent that is FDA-approved for the treatment of patients with glioblastoma multiforme. TMZ has shown modest activity in the treatment of lung cancer. Continuous low dose TMZ was studied in two single-arm phase II trials for patients with previously treated NSCLC. A trial of 47 patients explored TMZ given at a dose of 75 mg/m^2^/day for 6 out of 8 weeks (28). The ORR was 8% in 38 evaluable patients, with an additional 32% of patients achieving stable disease. Median event free survival was 49 days and median OS was 8.1 months with a projected 1-year survival rate of 30%. The dosing regimen was relatively well-tolerated, though 3 patients developed prolonged thrombocytopenia. Another trial of 31 patients with relapsed NSCLC utilized TMZ 75 mg/m^2^/day given 21 out of 28 days (29). In this study, the RR was only 6.5% though another 10% of patients achieved stable disease. Median TTP was 2.4 months and median OS was 3.3 months with a 1-year survival rate of 22.5%. There was one toxic death related to neutropenia, but the regimen was otherwise well-tolerated. An alternate dosing regimen was explored in a 25-patient study that included 12 patients with brain metastases and 13 patients without brain metastases (30). Patients received TMZ 200 mg/m^2^/day for 5 days in 28-day cycles. Grade 3–4 toxicities were primarily hematologic though nausea and lethargy were also observed (15–17%). No responses were seen with this regimen.

TMZ plays a more prominent potential role in the management of relapsed SCLC. A phase II study of TMZ 75 mg/m^2^/day for 21 out of 28 days included 64 patients with previously treated SCLC: 48 with platinum-sensitive relapse and 16 with platinum-refractory relapse (31). Sensitive disease was defined as relapse ≥ 60 days after platinum chemotherapy, whereas refractory disease was defined as progression within 60 days of initial therapy. In patients with sensitive disease, the RR was 23% and in patients with refractory disease, the RR was 13%. In patients with measurable brain metastases, the intracranial response rate was 38% and brain response was found to correlate with systemic response. The median duration of response was 3.5 months; the median OS was 5.8 months. Toxicities were generally mild, but grade 3–4 adverse events included thrombocytopenia (9%) and neutropenia (5%). The pulse dose regimen of TMZ 200 mg/m^2^/day on days 1–5 in 28-day cycles was also explored in patients with relapsed SCLC (32). This single arm phase II trial included 25 patients with previously treated SCLC (16 with platinum-sensitive relapse and 9 with refractory relapse). The RR was 12%; responses were observed in both sensitive and refractory relapse. The median TTP was 1.8 months and median OS was 5.8 months. Grade 3–4 thrombocytopenia was noted in 16% of patients (median duration 12 days), though cytopenias were reversible and did not limit further therapy. Although not FDA approved for the treatment of SCLC, temozolomide is a reasonable salvage option for patients with relapsed SCLC but has more modest activity in patients with relapsed NSCLC.

### Vinorelbine

Vinorelbine is a vinca alkaloid that inhibits microtubule polymerization (9). It is available in both parenteral and oral formulations, though efficacy seems greater with IV delivery. A phase II trial randomized 189 patients with advanced, treatment-naïve NSCLC in 2:1 fashion to receive oral vinorelbine 60 mg/m^2^ weekly (with potential escalation to 70 mg/m^2^ if well-tolerated) or standard IV vinorelbine 30 mg/m^2^ weekly (33). RR, the primary endpoint, favored the IV arm (13% with IV vinorelbine compared to 4% with oral). TTP was 23.9 weeks with IV vinorelbine and 16.6 weeks with oral vinorelbine. Survival also favored IV delivery with a median OS of 38.6 months in the IV vinorelbine arm vs. 30.3 months in the oral vinorelbine arm. In patients with a good performance status (Karnofsky Performance Status 80–100), outcomes were comparable with a median OS of 38.6 months with IV and 35.6 months with oral. As salvage therapy for NSCLC, oral vinorelbine has limited efficacy. A phase II study explored oral vinorelbine 60 mg/m^2^ once weekly in 20 patients with previously treated NSCLC (34). Though well-tolerated, there were no objective responses and the median time to progression was limited to 2 months.

While IV vinorelbine remains the standard, the schedule and tolerability are of concern for some patients. This led to the exploration of oral vinorelbine in special patient populations, where modest efficacy was observed. A single-arm study explored metronomic dosing of oral vinorelbine in 50 patients with advanced NSCLC who were not candidates for standard chemotherapy due to age, performance status, or comorbidities (35). Vinorelbine was given orally at a fixed dose of 30 mg three times a week. The RR was 8% with a median PFS of 2.7 months and a median OS of 7.3 months. Grade 3 adverse events were seen in 11% of patients (anemia, neutropenia, asthenia) with no grade 4 toxicity. In elderly patients, oral vinorelbine was relatively well-tolerated. A phase II study of oral vinorelbine 60 mg/m^2^ once weekly (with escalation to 80 mg/m^2^ weekly if well-tolerated) included 56 patients over age 70 with chemotherapy-naïve, advanced NSCLC (36). While grade 4 neutropenia was observed in 30% of patients, neutropenic fever was only seen in 1 patient. The RR was 11% with a median OS of 8.2 months. Another phase II trial included 43 elderly patients (age 70 or greater) with a poor performance status (Eastern Cooperative Oncology Group Performance Status 2–3) and advanced NSCLC (37). Oral vinorelbine was given at a dose of 60 mg/m^2^ on days 1–8 in 21-day cycles. The schedule was safe with only 1 case of grade 3 neutropenia (which spontaneously recovered) and no febrile neutropenia. There were no other grade 3 or higher adverse events. The RR was 18.6% with a median time to progression of 4 months. Median OS was 8 months with a 1-year survival rate of 37.2%. A more modern study randomized 165 patients with advanced NSCLC who were unfit for platinum-based chemotherapy to receive metronomic oral vinorelbine (50 mg fixed dose three times per week) or oral vinorelbine given 60 mg/m^2^ weekly (with escalation to 80 mg/m^2^) (38). The metronomic regimen was better tolerated with fewer adverse events including lower rates of neutropenia (11 vs. 52%) and febrile neutropenia (3.6 vs. 6.2%) with higher relative dose intensity (85 vs. 69%). Median PFS was comparable (4.3 months with metronomic vs. 3.9 months with weekly) as was median OS (7.1 and 7.6 months, respectively). The primary endpoint of PFS without grade 4 toxicity did favor the metronomic schedule.

A hybrid approach with IV and oral vinorelbine has been explored in combination with platinum therapy. A single-arm study described this approach in the adjuvant setting; 74 patients with completely resected stage IB-IIIA NSCLC suitable for chemotherapy received up to 4 cycles of IV carboplatin AUC 5 on day 1 with vinorelbine 25 mg/m^2^ IV on day 1 and 60 mg/m^2^ orally on day 8 in 21-day cycles (39). While the lack of a comparator arm makes interpretation challenging, dose delivery was high with 83.7% of patients completing all 4 planned cycles. The median disease-specific survival was 7.63 years, median OS was 5.9 years and the 5-year survival rate was 56.2%.

There is also activity with oral vinorelbine in patients with locally advanced NSCLC, though there is a lack of randomized data. A phase II trial combined oral vinorelbine with IV cisplatin in 54 patients with stage III NSCLC undergoing definitive radiation therapy (40). Patients received two cycles of induction therapy with IV cisplatin 80 mg/m^2^ on day 1 and oral vinorelbine 60 mg/m^2^ on days 1 and 8 (with escalation to 80 mg/m^2^ if well-tolerated) every 3 weeks. In the absence of progression, patients then received two additional cycles of cisplatin 80 mg/m^2^ on day 1 with oral vinorelbine 40 mg/m^2^ on days 1 and 8 every 3 weeks concurrently with radiotherapy (66 Gy in 6.5 weeks). Dose intensity was high for both vinorelbine and cisplatin in induction (86 and 93%) and during chemoradiation (97 and 98%). Grade 3–4 neutropenia was observed in 28% of patients with febrile neutropenia in 7% and grade 3 nausea in 11%. The RR was 54% with a median PFS of 12.5 months and a median OS of 23.4 months. The hybrid regimen was also explored in the neoadjuvant setting (41). In 58 patients with treatment-naïve, unresectable stage III NSCLC, cisplatin 60 mg/m^2^ was given on day 1 with weekly oral vinorelbine 50 mg/m^2^ for two cycles concurrently with radiation (66–70 Gy for 6.5–7 weeks) followed by either surgery (if a candidate) or consolidation cisplatin plus IV vinorelbine (with the option for surgery if then a candidate). After chemoradiation, the RR was 53.4%, the median PFS was 6.7 months and the median OS was 24.8 months. Overall, 29.3% of patients were downstaged and able to proceed to surgical resection with 7 patients achieving pathologic complete remission. Grade 3–4 neutropenia was seen in 11% of patients with only 1 case of febrile neutropenia.

The hybrid approach of combining oral vinorelbine with IV platinum appears comparable to other platinum doublets (42–44), but the use of oral vinorelbine is appealing to patients. A randomized cross-over trial included 61 patients with treatment-naïve, incurable NSCLC (45). Patients received 6 cycles of IV carboplatin AUC 5 plus vinorelbine on days 1 and 8 in 21-day cycles. Vinorelbine was given either IV 30 mg/m^2^ or orally 60 mg/m^2^. Patients were randomized to receive IV vinorelbine for cycles 1 and 2 then oral vinorelbine for cycles 3 and 4 or oral vinorelbine first followed by IV vinorelbine. Patients could then choose IV or oral vinorelbine for cycles 5 and 6. When given the option, after having received both IV and oral vinorelbine, 74% of patients preferred oral vinorelbine. There was no significant difference between the two strategies in RR; the overall RR was 23% with a median OS of 11.4 months. Apart from grade 3–4 leukopenia, which was more common with IV vinorelbine (52 vs. 10%), toxicities were comparable with the two approaches. When IV vinorelbine is indicated, oral vinorelbine may be a consideration, particularly as part of a hybrid regimen, but its role in the modern management of advanced NSCLC is limited.

### S-1

S-1 (tegafur/gimeracil/oteracil) is an oral fluoropyrimidine chemotherapeutic that consists of a 5-fluorouracil (5-FU) prodrug (tegafur), a dihydropyrimidine dehydrogenase inhibitor (gimeracil), and an orotate phosphoribosyltransferase inhibitor (oteracil) in a molar ratio of 1:0.4:1. Gimeracil prevents the degradation of active 5-FU, thereby enhancing systemic concentration of the active agent, and oteracil limits activation of 5-FU in the gastrointestinal tract, thereby mitigating mucosal damage and limiting side effects such as diarrhea and stomatitis. The bulk of data supporting the efficacy of S-1 in advanced NSCLC comes from Japan, where platinum plus S-1 combination chemotherapy is an approved first-line regimen for the treatment of advanced NSCLC (46). Data in support of this regimen comes from two phase III Japanese trials that demonstrated the non-inferiority of platinum plus S-1 compared to platinum plus taxane (47, 48).

S-1 is also approved as monotherapy in Japan, where it carries a recommendation for second- or further-line treatment in patients with advanced NSCLC and a performance status of 0–2 (46). Data supporting this regimen comes from the East Asia S-1 Trial, a phase III non-inferiority study conducted at 84 medical centers in China, Japan, Hong Kong, Singapore, and Taiwan (49). In this study, 1,154 patients with advanced NSCLC who had progressed on ≤ 2 lines of systemic chemotherapy including ≥1 platinum-based regimen, were randomized to treatment with docetaxel 75 mg/m^2^ every 3 weeks or S-1 80–120 mg (dosed by body surface area) twice per day on days 1–28 of a 6-week cycle. The ORR for S-1 and docetaxel were 8.3 and 9.9%, respectively. S-1 demonstrated non-inferiority to docetaxel with a median overall survival (OS) of 12.75 months compared to 12.52 months (HR 0.945; 95% CI, 0.833–1.073), respectively. Similar efficacy was observed regardless of gender, ethnicity, *EGFR* mutation status, or histology. In addition, patients randomized to the S-1 arm had significantly better global health quality of life scores compared to those randomized to docetaxel, with improvement in scores assessing chest pain, dyspnea, peripheral neuropathy, and alopecia. S-1 also demonstrated an encouraging toxicity profile; grade 3–4 toxicities were rare with decreased appetite (6.5%), diarrhea (6.3%), and neutropenia (5.4%) observed most frequently. Grade 3–4 toxicities were much more common in the docetaxel arm, the most common being neutropenia (47.7%), leukopenia (29.1%), and febrile neutropenia (13.4%). Multiple phase II single-arm Japanese studies have also demonstrated encouraging efficacy of S-1 as monotherapy in treatment-naïve elderly patients with advanced NSCLC, a population that frequently cannot tolerate platinum-doublet chemotherapy. Studies have shown ORR of 7.9 to 27.6% and median OS of 7.5 to 17.0 months in this population (50–53). The variation in these metrics is likely attributable to small sample sizes and variable dosing schemes employed in these trials.

The data on outcomes with S-1 in Western populations are far less robust. The largest published trial to date of S-1 monotherapy in the United States is a phase II single-arm multicenter study in which 57 patients with advanced NSCLC who had progressed after platinum-doublet chemotherapy were treated with S-1 30 mg/m^2^ twice per day on days 1–14 of a 21 day cycle (54). ORR was 7.1% with a median OS of 7.3 months. Gastrointestinal (GI) toxicities were frequently observed with any-grade nausea (54%), diarrhea (49%), fatigue (40%), and vomiting (39%) as the most common; 21% of patients experienced grade ≥3 diarrhea. This side-effect profile stands in stark contrast to that observed in Japanese patients, who had far less severe GI toxicity. One proposed reason for this difference is the variation in *CYP2A6* gene polymorphisms (responsible for tegafur metabolism) among Japanese and Caucasian individuals, which may also play a role in the discrepancy in efficacy reported in these populations (55). Variation in the prevalence of now actionable *EGFR* driver mutations and the known differences in disease biology may also have factored into the efficacy discrepancy between populations.

Taken together, multiple studies support the use of S-1 as treatment in the subsequent line setting for East Asian populations with advanced NSCLC. Relative benefits of this regimen include convenience and an improved toxicity profile compared to other salvage chemotherapies such as docetaxel. However, there is limited data to support the use of S-1 in Western nations. Furthermore, S-1 lacks FDA approvals in any malignancy, limiting its applicability in the United States.

### Capecitabine

Capecitabine is an oral prodrug that is metabolized to 5-FU through a 3-step enzymatic process, the final step of which utilizes the enzyme thymidine phosphorylase (TP), which is preferentially expressed in some tumor tissues (56). Capecitabine is FDA-approved for the treatment of colorectal and breast cancer and is active against a host of other cancers, primarily those of GI origin (57). However, capecitabine monotherapy has not been studied extensively in NSCLC. There are no published reports or trials of single-agent capecitabine in advanced NSCLC outside of a case report detailing a patient with NSCLC metastatic to brain with leptomeningeal carcinomatosis with complete response to capecitabine following cranial radiotherapy (58).

In Korea, capecitabine has been studied with IV irinotecan in NSCLC, demonstrating an ORR of 11.4% with median OS of 7.4 months in a phase II trial of 37 patients with previously treated advanced NSCLC (59). In a phase II trial of 53 treatment-naïve NSCLC patients, the combination achieved an ORR of 41.5% with median OS of 14.6 months (60). This regimen has not been studied in Western populations. An all oral regimen of oral irinotecan with capecitabine has been investigated, but to this point studies have not proceeded past the phase I dose-finding phase in advanced solid tumors (61, 62).

In thoracic oncology, capecitabine has thus far demonstrated the most promise when combined with temozolomide (CAPTEM) for the treatment of advanced bronchopulmonary neuroendocrine tumors. The major data in support of this regimen comes from the phase II randomized multicenter ECOG-ACRIN E2211 study in which 144 patients with advanced pancreatic neuroendocrine tumors were randomized to treatment with temozolomide 200 mg/m^2^ daily on days 1–5 (TMZ) or TMZ plus capecitabine 750 mg/m^2^ twice per day on days 1–14 of a 28 day cycle (CAPTEM) (63). CAPTEM demonstrated an improvement in PFS with a median PFS of 22.7 vs. 14.4 months. There was also an improvement in survival (OS HR 0.41, *p* = 0.012), though the CAPTEM regimen was more toxic with higher rates of grade 3/4 adverse events including neutropenia (13 vs. 4%), nausea/vomiting (8 vs. 0%), diarrhea (8 vs. 0%), and fatigue (8 vs. 1%). While prospective trial data for CAPTEM in bronchopulmonary neuroendocrine tumors are lacking, multiple small single-center retrospective analyses have supported the encouraging data from E2211, demonstrating ORR of 18–30%, disease control rates of 76–85%, and median OS of 30.4 to 68 months (64, 65). In addition, the toxicity profile of CAPTEM appeared quite favorable in these retrospective studies with the most common grade 3/4 adverse event, thrombocytopenia, occurring in 10–15% of patients. While not FDA approved, CAPTEM carries a category 2A NCCN recommendation for the treatment of locoregionally advanced and/or metastatic bronchopulmonary neuroendocrine tumors (66).

In summary, there is no data to support the use of capecitabine in the treatment of NSCLC. For advanced bronchopulmonary neuroendocrine tumors, however, CAPTEM is a promising regimen that should be considered if systemic cytoreductive therapy is warranted.

## Conclusion

Systemic therapy plays a critical role in the palliative management of advanced lung cancer. Selection of specific regimens always involves a balance of benefit and risk. The COVID-19 pandemic has introduced a new variable to that equation: the risk of traveling to an infusion center for IV therapeutics. Given this added risk, the risk:benefit ratio for any therapy must be carefully assessed and it is important to recall that in some specific instances, forgoing treatment could be appropriate. For patients who may benefit from therapy, selection of the specific regimen can now be influenced by route of administration and schedule. While most of the standard agents for NSCLC are administered intravenously, there are circumstances where an oral cytotoxic agent may be a comparable alternative and when all else is equal, the ability to receive therapy while staying at home is increasingly appealing in this new era.

## Author Contributions

All authors listed have made a substantial, direct and intellectual contribution to the work, and approved it for publication.

## Conflict of Interest

CK has received research grants (to institution) from Altor Bioscience, AstraZeneca, BMS, Debiopharm, Karyopharm, Novartis, Regeneron, and Tesaro, and is an advisory board member for Novartis. SL reports receiving commercial research grants (to institution) from Alkermes, AstraZeneca, Bayer, Blueprint, Bristol-Myers Squibb, Corvus, Genentech/ Roche, Lilly, Lycera, Merck, Merus, Molecular Partners, Pfizer, Rain Therapeutics, RAPT, Spectrum and Turning Point Therapeutics, and is a consultant/advisory board member for AstraZeneca, Boehringer Ingelheim, Bristol-Myers Squibb, Catalyst, Celgene, G1 Therapeutics, Genentech/Roche, Guardant Health, Janssen, Lilly, LOXO, MSD, Pfizer, PharmaMar, Regeneron, and Takeda. The remaining authors declare that the research was conducted in the absence of any commercial or financial relationships that could be construed as a potential conflict of interest.

## References

[1] Liang W Guan W Chen R Wang W Li J Xu K. Cancer patients in SARS-CoV-2 infection: a nationwide analysis in China. Lancet Oncol. (2020) 21:335–7. 10.1016/S1470-2045(20)30096-6 32066541 PMC7159000

[2] Passaro A Peters S Mok TSK Attili I Mitsudomi T de Marinis F. Testing for COVID-19 in lung cancer patients. Ann Oncol. (2020). [Epub ahead of print]. 10.1016/j.annonc.2020.04.002 32278879 PMC7144604

[3] Yu J Ouyang W Chua MLK Xie C. SARS-CoV-2 transmission in patients with cancer at a Tertiary Care Hospital in Wuhan, China. JAMA Oncol. (2020). [Epub ahead of print]. 10.1001/jamaoncol.2020.0980 32211820 PMC7097836

[4] Ueda M Martins R Hendrie PC McDonnell T Crews JR Wong TL. Managing cancer care during the COVID-19 pandemic: agility and collaboration toward a common goal. J Natl Compr Cancer Netw. (2020). 10.6004/jnccn.2020.7560 [Epub ahead of print]. 32197238

[5] Yuan M Huang L-L Chen J-H Wu J Xu Q. The emerging treatment landscape of targeted therapy in non-small-cell lung cancer. Signal Transduct Target Ther. (2019) 4:61. 10.1038/s41392-019-0099-9 31871778 PMC6914774

[6] Bristol-Myers Squibb VePesid® (etoposide) [package insert]. U.S. Food and Drug Administration website (2004). Available online at: https://www.accessdata.fda.gov/drugsatfda_docs/label/2002/019557s028lbl.pdf (accessed April, 2020).

[7] GlaxoSmithKline HYCAMTIN® (topotecan)[package insert]. U.S. Food and Drug Administration website (2007). Available online at: https://www.accessdata.fda.gov/drugsatfda_docs/label/2007/020981lbl.pdf (accessed April, 2020).

[8] Merck & Co TEMODAR® (temozolomide) [package insert]. U.S. Food and Drug Administration website (2016). Available online at: https://www.accessdata.fda.gov/drugsatfda_docs/label/2016/021029s031lbl.pdf (accessed April, 2020).

[9] Barletta G Genova C Rijavec E Burrafato G Biello F Sini C. Oral vinorelbine in the treatment of non-small-cell lung cancer. Expert Opin Pharmacother. (2014) 15:1585–99. 10.1517/14656566.2014.934224 24972635

[10] Taiho Pharmaceutical Co Teysuno®. European Public Assessment Reports. Available online at: https://www.ema.europa.eu/en/documents/product-information/teysuno-epar-product-information_en.pdf (accessed April, 2020).

[11] Genentech Inc XELODA (capecitabine) [package insert]. U.S. Food and Drug Administration website (2015). Available online at: https://www.accessdata.fda.gov/drugsatfda_docs/label/2015/020896s037lbl.pdf (accessed April, 2020).

[12] Horn L Mansfield AS Szczesna A Havel L Krzakowski M Hochmair MJ. First-line atezolizumab plus chemotherapy in extensive-stage small-cell lung cancer. N Engl J Med. (2018) 379:2220–9. 10.1056/NEJMoa1809064 30280641

[13] Paz-Ares L Dvorkin M Chen Y Reinmuth N Hotta K Trukhin D. Durvalumab plus platinum–etoposide versus platinum–etoposide in first-line treatment of extensive-stage small-cell lung cancer (CASPIAN): a randomised, controlled, open-label, phase 3 trial. Lancet. (2019) 394:1929–39. 10.1016/S0140-6736(19)32222-6 31590988

[14] Johnson DH Ruckdeschel JC Keller JH Lyman GH Kallas GJ Macdonald J. A randomized trial to compare intravenous and oral etoposide in combination with cisplatin for the treatment of small cell lung cancer. Cancer. (1991) 67:245–9. 10.1002/1097-0142(19910101)67:1+<245::aid-cncr2820671306>3.0.co;2-z 1845847

[15] Furuse K. [A phase II study of etoposide (NK 171) in small cell lung cancer–comparison of results between intravenous administration and oral administration]. Gan To Kagaku Ryoho. (1985) 12:2352–7. 3000299

[16] Miller AA Herndon JE Hollis DR Ellerton J Langleben A Richards F. Schedule dependency of 21-day oral versus 3-day intravenous etoposide in combination with intravenous cisplatin in extensive-stage small-cell lung cancer: a randomized phase III study of the Cancer and Leukemia Group B. J Clin Oncol. (1995) 13:1871–9. 10.1200/JCO.1995.13.8.1871 7636529

[17] Sundstrøm S Bremnes RM Kaasa S Aasebø U Hatlevoll R Dahle R. Cisplatin and etoposide regimen is superior to cyclophosphamide, epirubicin, and vincristine regimen in small-cell lung cancer: results from a randomized phase III trial with 5 years' follow-up. J Clin Oncol. (2002) 20:4665–72. 10.1200/JCO.2002.12.111 12488411

[18] Saxman S Loehrer PJ Logie K Stephens D Workman F Scullin D. Phase II trial of daily oral etoposide in patients with advanced non-small cell lung cancer. Invest New Drugs. (1991) 9:253–6. 10.1007/bf00176978 1664423

[19] Waits TM Johnson DH Hainsworth JD Hande KR Thomas M Greco FA. Prolonged administration of oral etoposide in non-small-cell lung cancer: a phase II trial. J Clin Oncol. (1992) 10:292–6. 10.1200/JCO.1992.10.2.292 1310104

[20] Estapé J Palombo H Sánchez-Lloret J Agustí C Grau JJ Daniels M. Chronic oral etoposide in non-small cell lung carcinoma. Eur J Cancer. (1992) 28A:835–7. 10.1016/0959-8049(92)90126-m 1326308

[21] Miller AA Niell HB Griffin JP. Phase II study of prolonged oral etoposide in combination with intravenous cisplatin in advanced non-small cell lung cancer. Lung Cancer. (1995) 12:59–65. 10.1016/0169-5002(94)00406-d 7600031

[22] von Pawel J Gatzemeier U Pujol JL Moreau L Bildat S Ranson M. Phase ii comparator study of oral versus intravenous topotecan in patients with chemosensitive small-cell lung cancer. J Clin Oncol. (2001) 19:1743–9. 10.1200/JCO.2001.19.6.1743 11251005

[23] Eckardt JR von Pawel J Pujol J-L Papai Z Quoix E Ardizzoni A. Phase III study of oral compared with intravenous topotecan as second-line therapy in small-cell lung cancer. J Clin Oncol. (2007) 25:2086–92. 10.1200/JCO.2006.08.3998 17513814

[24] Agelaki S Kontopodis E Kotsakis A Chandrinos V Bompolaki I Zafeiriou Z. A phase I clinical trial of weekly oral topotecan for relapsed small cell lung cancer. Cancer Chemother Pharmacol. (2013) 72:45–51. 10.1007/s00280-013-2167-0 23604531

[25] White SC Cheeseman S Thatcher N Anderson H Carrington B Hearn S. Phase II study of oral topotecan in advanced non-small cell lung cancer. Clin Cancer Res. (2000) 6:868–73. 10741709

[26] Ramlau R Gervais R Krzakowski M von Pawel J Kaukel E Abratt RP. Phase III study comparing oral topotecan to intravenous docetaxel in patients with pretreated advanced non–small-cell lung cancer. J Clin Oncol. (2006) 24:2800–7. 10.1200/JCO.2005.03.6491 16682727

[27] Jones S Thompson D Barton J Patton J Shipley D Greco FA. A randomized phase II trial of oral topotecan versus docetaxel in the second-line treatment of non-small-cell lung cancer. Clin Lung Cancer. (2008) 9:154–9. 10.3816/CLC.2008.n.023 18621625

[28] Adonizio CS Babb JS Maiale C Huang C Donahue J Millenson MM. Temozolomide in non-small-cell lung cancer: preliminary results of a phase II trial in previously treated patients. Clin Lung Cancer. (2002) 3:254–8. 10.3816/clc.2002.n.009 14662033

[29] Kouroussis C Vamvakas L Vardakis N Kotsakis A Kalykaki A Kalbakis K. Continuous administration of daily low-dose temozolomide in pretreated patients with advanced non-small cell lung cancer: a phase II study. Oncology. (2009) 76:112–7. 10.1159/000192586 19142045

[30] Dziadziuszko R Ardizzoni A Postmus PE Smit EF Price A Debruyne C. Temozolomide in patients with advanced non-small cell lung cancer with and without brain metastases. A phase II study of the EORTC Lung Cancer Group (08965). Eur J Cancer. (2003) 39:1271–6. 10.1016/s0959-8049(03)00234-x 12763216

[31] Pietanza MC Kadota K Huberman K Sima CS Fiore JJ Sumner DK. Phase II trial of temozolomide in patients with relapsed sensitive or refractory small cell lung cancer, with assessment of methylguanine-DNA methyltransferase as a potential biomarker. Clin Cancer Res. (2012) 18:1138–45. 10.1158/1078-0432.CCR-11-2059 22228633

[32] Zauderer MG Drilon A Kadota K Huberman K Sima CS Bergagnini I. Trial of a 5-day dosing regimen of temozolomide in patients with relapsed small cell lung cancers with assessment of methylguanine-DNA methyltransferase. Lung Cancer. (2014) 86:237–40. 10.1016/j.lungcan.2014.08.007 25194640 PMC4497567

[33] Hirsh V Desjardins P Needles BM Rigas JR Jahanzeb M Nguyen L. Oral versus intravenous administration of vinorelbine as a single agent for the first-line treatment of metastatic nonsmall cell lung carcinoma (NSCLC): a randomized phase II trial. Am J Clin Oncol. (2007) 30:245–51. 10.1097/01.coc.0000256103.21797.e5 17551300

[34] Rossi D Catalano V Alessandroni P Fedeli A Fedeli SL Giordani P. A phase II study of single-agent oral vinorelbine in patients with pretreated advanced non-small-cell lung cancer. Clin Lung Cancer. (2007) 8:382–5. 10.3816/clc.2007.n.019 17562239

[35] Banna GL Camerini A Bronte G Anile G Addeo A Rundo F. Oral metronomic vinorelbine in advanced non-small cell lung cancer patients unfit for chemotherapy. Anticancer Res. (2018) 38:3689–97. 10.21873/anticanres.12647 29848729

[36] Gridelli C Manegold C Mali P Reck M Portalone L Castelnau O. Oral vinorelbine given as monotherapy to advanced, elderly NSCLC patients: a multicentre phase II trial. Eur J Cancer. (2004) 40:2424–31. 10.1016/j.ejca.2004.07.028 15519515

[37] Camerini A Valsuani C Mazzoni F Siclari O Puccetti C Donati S. Phase II trial of single-agent oral vinorelbine in elderly (> or =70 years) patients with advanced non-small-cell lung cancer and poor performance status. Ann Oncol. (2010) 21:1290–5. 10.1093/annonc/mdp525 19914959

[38] Kowalski DM Morabito A Montanino A Bernabi R Grossi F Ramlau R Final results of randomized phase II trial of metronomic vs weekly oral vinorelbine as first-line chemotherapy in advanced NSCLC patients unfit to platinum-based chemotherapy: TEMPO LUNG. Ann Oncol. (2020) 30(suppl.5):v602–60. 10.1093/annonc/mdz260

[39] Kolek V Grygárková I Koubková L Skričková J Švecová J Sixtová D. Carboplatin with intravenous and subsequent oral administration of vinorelbine in resected non-small-cell-lung cancer in real-world set-up. PLOS ONE. (2017) 12:e0181803. 10.1371/journal.pone.0181803 28732018 PMC5521844

[40] Krzakowski M Provencio M Utracka-Hutka B Villa E Codes M Kuten A. Oral vinorelbine and cisplatin as induction chemotherapy and concomitant chemo-radiotherapy in stage III non-small cell lung cancer: final results of an international phase II trial. J Thorac Oncol. (2008) 3:994–1002. 10.1097/JTO.0b013e31818396cb 18758302

[41] Hsu P Chang JW Wang C Wu C Lin Y Wang C. Oral vinorelbine plus cisplatin with concomitant radiotherapy as induction therapy for stage III non-small cell lung cancer: results of a single-arm prospective cohort study. Thorac Cancer. (2019) 10:1683–91. 10.1111/1759-7714.13125 31276309 PMC6669803

[42] Tan EH Rolski J Grodzki T Schneider CP Gatzemeier U Zatloukal P. Global Lung Oncology Branch trial 3 (GLOB3): final results of a randomised multinational phase III study alternating oral and i.v. vinorelbine plus cisplatin versus docetaxel plus cisplatin as first-line treatment of advanced non-small-cell lung cancer. Ann Oncol. (2009) 20:1249–56. 10.1093/annonc/mdn774 19276396

[43] Reck M Macha H-N Del Barco S Cornes P Vaissière N Morand M. Phase II study of oral vinorelbine in combination with carboplatin followed by consolidation therapy with oral vinorelbine as single-agent in unresectable localized or metastatic non-small cell lung carcinoma. Lung Cancer. (2009) 64:319–25. 10.1016/j.lungcan.2008.10.014 19095327

[44] Martoni AA Melotti B Sperandi F Giaquinta S Piana E Pavesi L. Hybrid (intravenous and oral) administration of vinorelbine plus cisplatinum followed by oral vinorelbine as first-line therapy of advanced non-small cell lung cancer: a phase II study. Lung Cancer. (2008) 60:387–92. 10.1016/j.lungcan.2007.11.006 18160123

[45] Jensen LH Osterlind K Rytter C. Randomized cross-over study of patient preference for oral or intravenous vinorelbine in combination with carboplatin in the treatment of advanced NSCLC. Lung Cancer. (2008) 62:85–91. 10.1016/j.lungcan.2008.02.009 18372075

[46] Akamatsu H Ninomiya K Kenmotsu H Morise M Daga H Goto Y. The Japanese Lung Cancer Society Guideline for non-small cell lung cancer, stage IV. Int J Clin Oncol. (2019) 24:731–70. 10.1007/s10147-019-01431-z 31049758 PMC6545178

[47] Kubota K Sakai H Katakami N Nishio M Inoue A Okamoto H. A randomized phase III trial of oral S-1 plus cisplatin versus docetaxel plus cisplatin in Japanese patients with advanced non-small-cell lung cancer: TCOG0701 CATS trial. Ann Oncol. (2015) 26:1401–8. 10.1093/annonc/mdv190 25908605 PMC4478975

[48] Okamoto I Yoshioka H Morita S Ando M Takeda K Seto T. Phase III trial comparing oral S-1 plus carboplatin with paclitaxel plus carboplatin in chemotherapy-naïve patients with advanced non-small-cell lung cancer: results of a west Japan oncology group study. J Clin Oncol. (2010) 28:5240–6. 10.1200/JCO.2010.31.0326 21079147

[49] Nokihara H Lu S Mok TSK Nakagawa K Yamamoto N Shi YK. Randomized controlled trial of S-1 versus docetaxel in patients with non-small-cell lung cancer previously treated with platinum-based chemotherapy (East Asia S-1 Trial in Lung Cancer). Ann Oncol. (2017) 28:2698–706. 10.1093/annonc/mdx419 29045553 PMC5834128

[50] Goto H Okano Y Machida H Hatakeyama N Ogushi F Haku T. Phase II study of tailored S-1 monotherapy with a 1-week interval after a 2-week dosing period in elderly patients with advanced non-small cell lung cancer. Respir Investig. (2018) 56:80–6. 10.1016/j.resinv.2017.09.003 29325686

[51] Nishiyama O Taniguchi H Kondoh Y Takada K Baba K Saito H. Phase II study of S-1 monotherapy as a first-line treatment for elderly patients with advanced nonsmall-cell lung cancer: the Central Japan Lung Study Group trial 0404. Anticancer Drugs. (2011) 22:811–6. 10.1097/CAD.0b013e3283440231 21317767

[52] Kasai T Nakamura Y Fukuda M Kitazaki T Nagashima S Takatani H. A phase II study of S-1 for previously untreated elderly patients with advanced non-small cell lung cancer. Chemotherapy. (2016) 61:93–8. 10.1159/000441486 26606381

[53] Shiroyama T Kijima T Komuta K Yamamoto S Minami S Ogata Y. Phase II tailored S-1 regimen study of first-line chemotherapy in elderly patients with advanced and recurrent non-small cell lung cancer. Cancer Chemother Pharmacol. (2012) 70:783–9. 10.1007/s00280-012-1958-z 22960985

[54] Govindan R Morgensztern D Kommor MD Herbst RS Schaefer P Gandhi J. Phase II trial of S-1 as second-line therapy in patients with advanced non-small cell lung cancer. J Thorac Oncol. (2011) 6:790–5. 10.1097/JTO.0b013e3182103b51 21325974

[55] Nakajima M Fukami T Yamanaka H Higashi E Sakai H Yoshida R. Comprehensive evaluation of variability in nicotine metabolism and CYP2A6 polymorphic alleles in four ethnic populations. Clin Pharmacol Ther. (2006) 80:282–97. 10.1016/j.clpt.2006.05.012 16952495

[56] Miwa M Ura M Nishida M Sawada N Ishikawa T Mori K. Design of a novel oral fluoropyrimidine carbamate, capecitabine, which generates 5-fluorouracil selectively in tumours by enzymes concentrated in human liver and cancer tissue. Eur J Cancer. (1998) 34:1274–81. 10.1016/s0959-8049(98)00058-6 9849491

[57] Walko CM Lindley C. Capecitabine: a review. Clin Ther. (2005) 27:23–44. 10.1016/j.clinthera.2005.01.005 15763604

[58] Paydas S Bicakci K Yavuz S. Dramatic response with capecitabine after cranial radiation to the brain parenchymal and leptomeningeal metastases from lung cancer. Eur J Intern Med. (2009) 20:96–9. 10.1016/j.ejim.2008.04.015 19237101

[59] Han J-Y Lee DH Kim HY Kim E-A Lee JJ Ju SY. A Phase II study of weekly irinotecan and capecitabine in patients with previously treated non-small cell lung cancer. Clin Cancer Res. (2003) 9:5909–14. 14676114

[60] Han J-Y Lee DH Lee SY Park CG Kim HY Lee HG. Phase II study of weekly irinotecan plus capecitabine for chemotherapy-naive patients with advanced nonsmall cell lung carcinoma. Cancer. (2005) 104:2759–65. 10.1002/cncr.21563 16294344

[61] Soepenberg O Dumez H Verweij J Semiond D deJonge MJA Eskens FALM. Phase I and pharmacokinetic study of oral irinotecan given once daily for 5 days every 3 weeks in combination with capecitabine in patients with solid tumors. J Clin Oncol. (2005) 23:889–98. 10.1200/JCO.2005.01.008 15681535

[62] Kümler I Eefsen RL Sørensen PG Theile S Fullerton A Nielsen PG. An open label phase 1 study evaluation safety, tolerability, and maximum tolerated dose of oral administration of irinotecan in combination with capecitabine. Cancer Chemother Pharmacol. (2019) 84:441–6. 10.1007/s00280-019-03819-0 30949758

[63] Kunz PL Catalano PJ Nimeiri H Fisher GA Longacre TA Suarez CJ A randomized study of temozolomide or temozolomide and capecitabine in patients with advanced pancreatic neuroendocrine tumors: a trial of the ECOG-ACRIN Cancer Research Group (E2211). J Clin Oncol. (2018) 36:4004 10.1200/JCO.2018.36.15_suppl.4004 PMC9995105 36260828

[64] Papaxoinis G Kordatou Z McCallum L Nasralla M Lamarca A Backen A. Capecitabine and temozolomide in patients with advanced pulmonary carcinoid tumours. Neuroendocrinology. (2019) [Epub ahead of print]. 10.1159/000502864 31437838

[65] Al-Toubah T Morse B Strosberg J. Capecitabine and temozolomide in advanced lung neuroendocrine neoplasms. Oncologist. (2020) 25:e48–52. 10.1634/theoncologist.2019-0361 31455747 PMC6964126

[66] National Comprehensive Cancer Network Neuroendocrine and Adrenal Tumors (Version 1.2019) (2019). Available online at: https://www.nccn.org/professionals/physician_gls/pdf/neuroendocrine_blocks.pdf (accessed April, 2020).

